# The Children's Forgiveness Card Set: Development of a Brief Pictorial Card-Sorting Measure of Children's Emotional Forgiveness

**DOI:** 10.3389/fpsyg.2021.628152

**Published:** 2021-03-29

**Authors:** Emma Kemp, Peter Strelan, Rachel Margaret Roberts, Nicholas R. Burns, Kelly Lynn Mulvey

**Affiliations:** ^1^Flinders Centre for Innovation in Cancer, Flinders University, Adelaide, SA, Australia; ^2^School of Psychology, University of Adelaide, Adelaide, SA, Australia; ^3^Department of Psychology, North Carolina State University, Raleigh, NC, United States

**Keywords:** apology, measurement, children, forgiveness, pictorial cards

## Abstract

Friendships have important influences on children's well-being and future adjustment, and interpersonal forgiveness has been suggested as a crucial means for children to maintain friendships. However, existing measures of preadolescent children's forgiveness are restricted by developmental limitations to reporting emotional responses via questionnaire and inconsistent interpretations of the term “forgive.” This paper describes development and testing of concurrent and discriminant validity of a pictorial measure of children's emotional forgiveness, the Children's Forgiveness Card Set (CFCS). In Study 1, 148 Australian children aged 8–13 years (*M* = 10.54, *SD* = 1.35) responded to a hypothetical transgression in which apology was manipulated and completed the CFCS and extant measures of forgiveness and socially desirable responding. Following an exploratory factor analysis to clarify the structure of the CFCS, the CFCS correlated moderately with other forgiveness measures and did not correlate with socially desirable responding. Apology predicted CFCS responding among older children. In Study 2 an exploratory factor analysis broadly replicated the structure of the CFCS among a sample of *N* = 198 North American children aged 5–14 years (*M* = 9.39 years, *SD* = 1.67). We also fitted an exploratory bi-factor model to the Study 2 data which clarified which cards best measured general forgiveness, or positive or hostile aspects of responding to transgressions. Apology once again predicted the CFCS, this time regardless of age. The CFCS appears a potentially valid measure of children's emotional forgiveness. Potential applications and differences between explicit and latent forgiveness in children are discussed.

## Introduction

Childhood is recognized as a crucial time for the development of positive attributes such as social competence and self-esteem, and relationships with peers are regarded as important influences on such development, as well as being valued by the child (e.g., Bagwell et al., 1998). Accordingly, children's ability to maintain peer relationships and resolve conflicts is important for childhood well-being and for children's development into competent, well-functioning adults.

One recognized way of resolving conflict and maintaining valued relationships is through interpersonal forgiveness (McCullough, 2008). The psychological aspects of interpersonal forgiveness among adults are now well-established, having been studied in a range of contexts, including counseling (e.g., Wade et al., 2014), psychoanalysis (e.g., Mucci, 2018), social and intimate relations (e.g., Fehr et al., 2010), justice (e.g., Strelan, 2018), the workplace (Bradfield and Aquino, 1999), and groups (e.g., Wenzel, 2019). Forgiving has been conceptualized as a coping response (Strelan, 2019), the culmination of a process in which hurt individuals transition from feelings of anger and resentment to taking an approach-oriented stance toward transgressors (McCullough et al., 2000). Interpersonal forgiveness is generally agreed to consist of cognitive, emotional and motivational aspects and may include behavioral indicators; thus it comprises both intrapersonal (Worthington, 2001) and interpersonal (McCullough et al., 1997) dimensions. As a context-specific response, forgiveness is distinguished from *forgivingness* (the disposition to be forgiving). Further, forgiveness occurs at both decisional and emotional levels (Worthington et al., 2007).

## Forgiveness in Children

While forgiving is argued to be potentially beneficial to children (Denham et al., 2005), little published research examines forgiveness in preadolescent children (van der Wal et al., 2016). Indeed, Worthington and Wade (2019) summary of the state of forgiveness research concludes that “research to understand the development of forgiveness in children is… still scant” (p. 348). Forgiveness is often regarded as requiring the ability to attend to, regulate, and repair emotional responses, and to therefore necessitate abstract thought and emotional management skills commonly accepted as only developing in adolescence (e.g., Rizkalla et al., 2008; Christensen et al., 2011). However, children are able to intuit and interpret emotions, and forgiving includes replacing negative other-oriented with positive other-oriented emotions (Worthington et al., 2007). Thus, it is appropriate to examine forgiveness in preadolescent children, at least to the extent that forgiveness is measured primarily in terms of emotion-transformation (Worthington, 2006) that may be manifested behaviorally (e.g., McCullough et al., 1997).

### The Need for Alternative Measures of Children's Forgiveness

One significant barrier to conducting studies of children's forgiveness pertains to measurement. In contrast to the range of forgiveness measures available for use with adult respondents, forgiveness measures developed specifically for children and young adolescents appear limited to one latent questionnaire [Enright Forgiveness Inventory for Children (EFI-C); Enright, 2000], single-item measures that ask children how much they would “forgive” (e.g., Darby and Schlenker, 1982) and narrative tasks that involve asking children to recall a transgression and how they responded to that transgression (Wainryb et al., 2019). Alternatively, studies appropriate questionnaires developed for adults or adolescents (e.g., Hui and Chau, 2009). Problematically, questionnaire and single-item measures of forgiveness are already recognized as having limitations with adult respondents; but they may be particularly inappropriate for measuring preadolescent children's forgiveness due to significant developmental differences between preadolescents and adults.

#### General Limitations of Self-Report Measures

Single-item measures are arguably limited by reliance on a shared conceptualization of forgiveness between respondent and researcher, at odds with studies suggesting that lay conceptualizations of forgiveness are confounded with concepts generally excluded from academic understandings such as accepting, forgetting, and reconciling (e.g., Lawler-Row et al., 2007). Meanwhile, questionnaire measures rely on respondents being willing and able to respond according to their underlying motivations, and are thus susceptible to response biases. For example, forgiveness questionnaires may be influenced by socially desirable response bias, because forgiveness is viewed as a prosocial and therefore valued response (Lawler-Row et al., 2007) or, alternatively, a weak and therefore undesirable act (e.g., Strelan et al., 2017). Self-reports of forgiveness may also be limited by inability to report on subconscious responses, since self-reports assume a level of cognitive involvement and self-reflection (Greenwald and Banaji, 1995), contrary to studies suggesting forgiveness may occur at the automatic or subconscious level (e.g., Karremans and Aarts, 2007).

Overall, reliance on questionnaire and single-item measures in forgiveness research has led to concerns over mono-method bias, whereby inherent error variance may be mistakenly accepted as legitimate forgiveness-related variance (Hoyt and McCullough, 2005). Such concerns have led to recommendations for a multimodal approach to forgiveness research (e.g., Hoyt and McCullough, 2005) with more recent studies employing implicit (Goldring and Strelan, 2017) and behavioral measures (e.g., Carlisle et al., 2012). However, little research has examined alternative measures of forgiveness for preadolescents, despite the possibility that children's developmental status may mean traditional self-report measures are particularly limited for child respondents.

#### Limitations of Existing Measures for Children

Although single-item Likert-type measures of forgiveness have been used with preadolescents (e.g., Darby and Schlenker, 1982), few studies examine preadolescents' actual understanding of terms such as “forgive” and “forgiveness” in order to establish what a question asking children about “forgiving” might actually be measuring. Even fewer studies assess children's everyday understandings of forgiveness terms in an inductive (“bottom-up”) style, rather than comparing children's understandings to pre-existing (i.e., adult) frameworks. Findings on children's everyday understandings of forgiveness are therefore sparse. However, children do appear to understand forgiveness as related to or contingent upon apology (Enright et al., 1989; Kemp et al., 2009). For example, one study found approximately half the sample (aged 9–12) confused forgiveness with apology at some point during an interview (Kemp et al., 2009). Children's responses to single-item forgiveness measures may therefore reflect overt responses to or confusion with apology, rather than the shift in internal motivations implied by emotional forgiveness.

Self-report latent questionnaires may also be more problematic for preadolescents than for adolescents or adults. Researchers in child assessment have noted that age and cognitive development are likely to influence the accuracy of children's self-reports, with age-related requirements for children to accurately respond to self-report measures including that the child must have achieved sufficient reading and comprehension levels to accurately complete items (Kazdin and Petti, 1982). While reading difficulties may be overcome by verbal administration of questionnaires, verbally administered measures may increase socially desirable response biases as children may feel pressured to please the person administering the questionnaire (e.g., a researcher or teacher), who may be reasonably perceived by the child as an authority figure (Eiser et al., 2000; Barker and Weller, 2003). Further, children's interpretation of the *meaning* of items may vary depending on stages of cognitive development, mental age and formal thought processes (Kazdin and Petti, 1982). Thus, given that late childhood and adolescence are times of important developmental change in cognition and verbal capacities (Soto et al., 2008), some preadolescent children of any given age may have no difficulty in accurately completing a verbal self-report measure, however, others may have considerably more difficulty.

Children's ability to validly self-report on their mental and emotional states also appears to depend in part on what characteristics children are asked to report on. While young children are better able to report objectively on concrete events or experiences (e.g., violent experiences; Luby et al., 1999), they appear less able to report on their own personal characteristics, particularly when such characteristics could be associated with a degree of social desirability (e.g., self-esteem; see Luby et al., 1999). Soto et al. (2008) argue that a range of developmental differences, including preadolescents' less mature verbal comprehension skills, less developed ability for abstract thought including the ability to reflect consistently on the self, and less developed ability to reflect on internal, emotional processes and quantify them verbally, make accurate responding on self-report questionnaires assessing emotion-based phenomena particularly challenging for preadolescents compared to either adolescents or adults.

Difficulty reporting on emotions may also compound the potential for children to respond to self-report formats with response biases. For example, in one study children aged five to 12 responded with more prevalent extreme scores as tasks became more subjective and emotion-focused (i.e., questionnaire measures of emotion compared to questionnaire measures of physical judgments); while this tendency was more pronounced for younger children, it existed to some degree across all age groups in the sample (Chambers and Johnston, 2002).

Finally, lack of familiarity with self-report questionnaires may also increase the likelihood of socially desirable responses (Soto et al., 2008). Although the EFI-C (Enright, 2000) attempts to address comprehension difficulties by employing simple vocabulary and a visual aid to guide children's responses, examination of potential differences between responses on self-report questionnaires and alternative measures requires prior development of valid alternative measures.

#### Child-Focused Measurement of Forgiveness

Aside from experiencing difficulty completing traditional questionnaire measures, children may lack the motivation to complete them accurately as they may find them boring or intimidating (Barker and Weller, 2003). Researchers have sought to enhance the meaning and precision of children's measures by more thoroughly considering children's perspectives during the research process, arguing that research often fails to address whether traditional measures based on adult-identified criteria are meaningful to children (e.g., Barker and Weller, 2003; Fattore et al., 2007). Child-focused measurement therefore attempts to include aspects that children identify as important to the construct in question, and to use approaches other than traditional questionnaires (e.g., Eiser et al., 2000; Fattore et al., 2007). Arguably, because children's experiences of forgiveness may differ from adult experiences, measurement of children's forgiveness could benefit from similar effort being invested in designing more child-friendly, child-relevant measures of forgiveness.

#### Behavioral Measures

One possible alternative to self-report is to employ behavioral measures, as with adult samples (e.g., Carlisle et al., 2012). For example, one study employed children's prosocial responses toward an offending classmate (credits allocated toward receipt of a gift) as a behavioral indicator of forgiveness (van der Wal et al., 2016), while other research has examined distributing resources to transgressors and non-transgressors as a measure of forgiveness (Oostenbroek and Vaish, 2019). However, behavioral measures may indicate motivations other than forgiveness *per se*, for example, general prosocial tendencies.

Laboratory-based behavioral measures may also be limited in the ability to generalize to forgiveness in real-life situations. For ethical reasons, laboratory transgressions are weaker than real-life transgressions (Carlisle et al., 2012) and usually involve transgressions by strangers or authority figures (i.e., researchers), and thus may not generalize to children's real life interactions. Arguably, given forgiveness is at least partly an intra-psychic process (Worthington et al., 2007), behaviors can corroborate but cannot replace self-report measures; self-report and behavioral measures have even been found to be differentially predicted, suggesting distinct mechanisms (Carlisle et al., 2012). Further alternatives are therefore needed to assess *intrapersonal* aspects of children's forgiveness.

#### Other-Report Measures

Another alternative is other-report measurement, typically completed by parents or other adults; however, other-report measures have been suggested more appropriate for use with externalizing behaviors, rather than typically “less observable” internalizing behaviors (Eiser et al., 2000). They are arguably also inappropriate for measuring “less observable” (i.e., intrapersonal) aspects of forgiveness. Thus, other-report measures may supplement but cannot replace children's own reports of emotional forgiveness.

### Development of an Alternative Measure

As the above review indicates, research on preadolescent children's forgiveness would benefit from a child-friendly, self-report measure of emotional forgiveness that is not a questionnaire. This article describes the development of one such measure, the Children's Forgiveness Card Set (CFCS), and initial testing of this measure's validity.

Given the limitations of single-item and questionnaire measures, the ideal alternative measure of preadolescent children's forgiveness would be easy to use, require minimal reading, writing and verbal expression, and take account of children's own everyday experiences of forgiveness. Existing self-report alternatives to written or verbal questionnaires in child assessment include pictorial questionnaires (e.g., Children's Critical Illness Impact Scale; Rennik et al., 2008), sorting of cards into categories (e.g., Family Relations Test; Anthony and Bene, 1957), and forced-choice selection of responses depicted pictorially on cards (e.g., Challenging Situations Task; Denham and Bouril, 1994). For the new measure, these approaches were combined such that it consisted of a task in which children sorted illustrated cards depicting child-identified forgiving and unforgiving responses.

However, simply sorting cards might obtain extreme scores (i.e., all forgiving, or all unforgiving responses). Therefore, a response scale consisting of a 10 centimeter line with a cross at one end and a tick at the other (maintaining the cards' non-verbal nature) was added to each card; children responded on the CFCS by indicating how much they did or did not endorse the response depicted by marking the line, in addition to sorting cards.

### Developing Illustrations and Pilot Testing

Illustrations for the new measure were developed from interviews examining children's everyday understandings of forgiveness (Kemp et al., 2009). Emotional and behavioral indicators of forgiveness were selected as possible items if they (a) directly described forgiving or not forgiving and (b) did not simply describe forgiveness as a response to apology. Terms with similar meanings (e.g., angry, mad) or that could be represented by the same illustration (e.g., being friendly, saying “hi”) were reduced to one item. This process originally identified 16 items (see Table 1) which were then illustrated, representing forgiving emotional responses, forgiving behavioral responses, unforgiving emotional responses, and unforgiving behavioral responses, consistent with forgiveness conceptualized as a suite of such responses (e.g., Worthington et al., 2007).

**Table 1:** Reduced terms representing potential items for a new forgiveness measure.

**Feelings**	**Behaviors**
**Forgiving**	
Good (OK, better, awesome)	Friendly (body language, respect)
Happy (glad, satisfaction, relieved)	Playing/hanging out (friends again, talking nicely with each other again)
Warm (loving)	Helping (do nice things)
Joyful (liberating, free)	Moving on (get over it, give another chance, act like it never happened)
**Unforgiving**	
Hate	Arguing/fighting
Angry	Not talking/listening
Upset	Ignoring
Weird/confused	“Get away from me” (“don't touch me”)

Illustrations were simple, to facilitate interpretation and universality; emotions and behaviors were depicted using cartoon-like faces or figures, and all illustrations were presented as black lines on a white background without shading.

We tested face validity of illustrations in two ways. First, illustrations were reviewed for interpretability and suitability by a panel of three academic psychologists (including one clinical child psychologist), and four PhD (psychology) candidates undertaking forgiveness research.

Second, we gave the Card Set to two separate samples of children. In the first, 12 children (six girls) aged 9-12 years (*M* = 10, *SD* = 0.58) provided qualitative interpretations of each illustration and suggested how to more clearly depict intended responses. Six cards were identified as difficult to interpret and changes to these cards were made based on the more salient suggestions. A second sample of 24 children (15 girls) aged 9–10 years (*M* = 9, *SD* = 0.38) sorted the new set of cards according to whether they thought illustrations represented a forgiving or unforgiving response, or a response unrelated to forgiveness. Two cards (“ignoring” and “weird/confused”) were sorted into an opposite category by four and three children, respectively (nonetheless, we retained these cards, given that the first sample of children and the adult experts agreed they were “unforgiving” cards).

### Study 1

Study 1 had four aims. First, examine the structure of the CFCS via exploratory factor analysis (EFA). Second, assess construct validity of the CFCS as an indicator of children's forgiving responses by examining correlations between the CFCS and existing measures of children's forgiveness and a measure of socially desirable responding. Third, test incremental validity of the CFCS through the extent to which it explained variance in forgiveness behavior over and beyond an existing measure of children's forgiveness. Fourth, test the extent to which scores on the CFCS could be differentiated by a well-established predictor of transgression-specific forgiveness, apology. We now elaborate on the decisions underlying inclusion of extant measures and approaches, and our hypotheses.

Three existing measures of forgiveness were employed to test construct validity: a single-item explicit measure, a latent questionnaire measure, and a measure of relationship restoration.

Regardless of the hypothesized shortcomings of single-item explicit measures, because the CFCS was based on children's descriptions of “forgiving,” responses on the CFCS were expected to share a significant amount of variance with responses to the same transgression on a single-item explicit measure.

The CFCS was also expected to correlate with a latent measure of forgiveness. The EFI-C (Enright, 2000) was used, as it was designed for children aged 6–12 years, and uses simple language likely to be familiar to children (e.g., happy, bad) and a four-point response scale accompanied by a red circle/green circle visual aid. Further, the EFI-C consists of three subscales assessing cognitive, behavioral and emotional aspects of forgiveness, allowing for separation of emotion-based, behavioral, and cognitive responses. We expected the CFCS to be positively associated with each subscale, with the strongest correlation likely to be with the emotion-based subscale.

In addition, the CFCS was expected to be positively correlated with a measure of relationship restoration, an outcome of forgiveness often observed in forgiveness research among adults (for a meta-analysis see Fehr et al., 2010) and indicated in interviews with children who understood forgiveness as meaning a friendship is “back to normal” (Kemp et al., 2009).

Participants responded to a hypothetical transgression (described below) in which offender apology was manipulated. Apology is one of the best predictors of adult forgiveness [for a meta-analysis see Fehr et al. (2010)], with some evidence that it also predicts apology in children (e.g., Darby and Schlenker, 1982). Meanwhile, we employed a hypothetical transgression because scenario studies avoid the ethical limitations of either presenting children with a serious laboratory-contrived transgression (while also avoiding a transgression so mild that it did not present an ecologically valid offense), or asking children to recall a potentially distressing real-life transgression (e.g., bullying, abuse). Additionally, hypothetical scenarios do not require children to recall a transgression in sufficient detail to accurately report their responses. Finally, a hypothetical transgression offers a useful comparison with existing research, as hypothetical scenarios are commonly used in studies of children's social responses, including forgiveness (Darby and Schlenker, 1982; Denham et al., 2005) and responses to apology (e.g., Smith et al., 2010).

## Method

### Participants

There were 148 children (78 boys, 70 girls) aged 8–13 (*M* = 10.54, *SD* = 1.35), recruited from eight public primary schools in metropolitan Adelaide, South Australia. In terms of power, our aim was to collect as many participants as possible during a semester.

Ethical considerations prevented us from collecting data on individual participants' race and SES. However, we are able to provide some SES-relevant information about the schools from which participants were recruited. The South Australian government has developed an “Index of Educational Disadvantage,” which comprises measures of parental economic resources, parental education and occupation, Aboriginality, and student mobility. Schools are ranked on the index, where 1 = most disadvantaged and 7 = least disadvantaged. Participating schools in the present study ranged from category 2 to 7, with the mean ranking 5.5. Although we sampled schools from all but the lowest level of disadvantage, effectively the sample was comprised primarily of children from middle to higher level socioeconomic backgrounds. One may also presume that there were relatively few children from non-English speaking backgrounds [finally, it may be noted that pilot study children came from schools ranked 6 (pilot sample 2) and 5 (pilot sample 3)].

### Materials

#### Hypothetical Scenario: Manipulation of Apology

Students were presented with a hypothetical scenario describing a primary school-aged child being transgressed against by his/her best friend, who tells the child's embarrassing secret. Characters in the scenario were gender-matched to the respondents. In the apology manipulation, the best friend apologized, looked sad, and stated he/she felt bad and would do whatever he/she could to make up for the transgression. In the no apology manipulation, the best friend said nothing about the transgression and acted like nothing had happened. In order to assist children's understanding, the hypothetical transgression was accompanied by illustrations.

#### Background Variables and Manipulation Checks

Because severity is a key predictor of forgiveness that may also impact on the effectiveness of apologies (Fehr and Gelfand, 2010), perceived severity of the transgression was assessed as a potential covariate using a single item, “How bad is what happened in the story?” Response options (accompanied by illustrations) were “not bad at all” (happy face), “a bit bad” (slightly unhappy face), and “really bad” (very unhappy face).

#### Apology Manipulation Check

The *apology manipulation check* was a single item assessing perceived transgressor remorse; “How sorry do you think (transgressor) felt about what happened?” (“*not sorry at all*” (happy face), “*a bit sorry”* (slightly unhappy face), and “*very sorry*” (very sad face).

#### The CFCS

**Figure 1:**
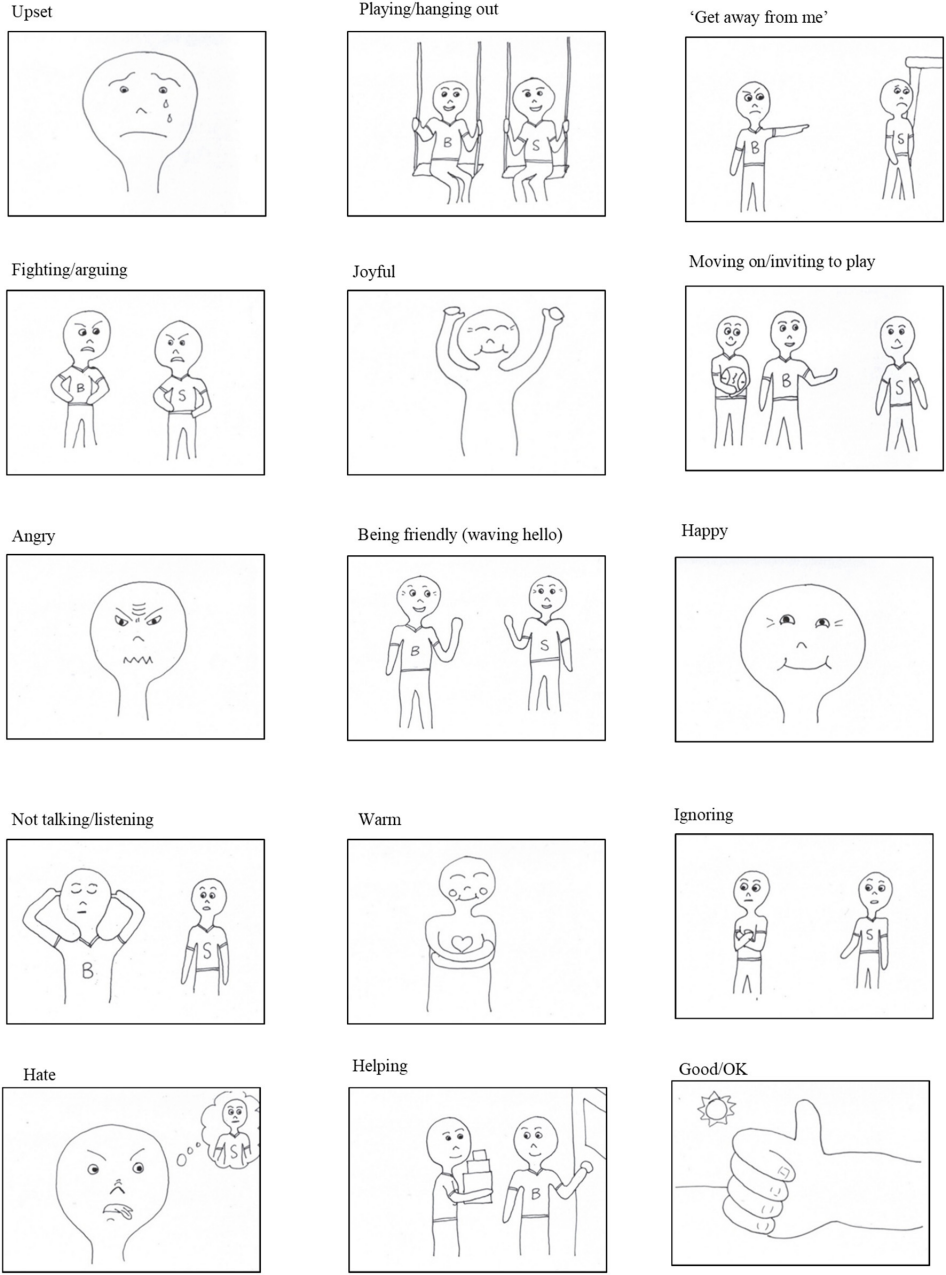
Illustrations included in the final version of the Children's Forgiveness Card Set. Each illustration is presented on a separate card, depicted above a 10 centimeter line with a cross illustrated at the left hand (0 cm) anchor and a tick illustrated at the right hand (10 cm) anchor of the line.

Following EFA (see Results section), the final version of the CFCS consisted of 15 illustrated cards (see Figure 1). On each card a 10 centimeter line with a cross at one end and a tick at the other enabled children to indicate the strength with which they would *feel or feel like acting* in the way depicted if they were the child transgressed against in the scenario. Cards were provided in a large envelope, accompanied by two smaller envelopes into which children sorted cards, one marked with a tick (for “*ways you would feel, or would feel like acting”*) and the other with a cross (for “*ways you wouldn't feel, or wouldn't feel like acting*”). Children responded by making a mark on a line *and* by sorting cards; thus the CFCS was scored in two ways. First, positive cards sorted into the “*would feel*” category and negative cards sorted into the “*wouldn't feel*” category were scored +1 while positive cards sorted in to the “*wouldn't feel*” category and negative cards sorted in to the “*would feel*” category were scored −1; scores were then summed to produce a Sort Task score. Second, the distance from the left anchor at which children marked the 10 centimeter line (thus effectively an 11 point scale from 0 to 10) was measured; negative cards were reverse-scored by subtracting this distance from 10 meaning higher values represented more forgiving responses regardless of whether the card was positive or negative; scores were then summed to produce the Line Task score.

#### EFI-C

The EFI-C (Enright, 2000) is a 30-item questionnaire measure of interpersonal forgiveness designed for children aged six to 12 years. Three subscales each consisting of 10 items assess forgiveness in terms of feelings (e.g., “*I feel happy toward him/her*”), behaviors (e.g., “*I am a friend to him/her*”) and thoughts (e.g., “*I think good thoughts about him/her*”). Children respond to each item on a four point scale (*1* = *NO!, 2* = *a little bit no, 3* = *a little bit yes, 4* = *YES!*), with an accompanying visual aid consisting of a large red, small red, small green, and large green circle. Internal consistency reliability in the current sample (Cronbach's α) was 0.78 for the Feelings subscale, 0.84 for the Behaviors subscale and 0.88 for the Thoughts subscale.

#### Single-Item Forgiveness

Single-item forgiveness was measured using an additional item of the EFI-C, “*Have you forgiven [transgressor]*,” using the same response scale as described for EFI-C subscales.

#### Relationship Restoration

*Relationship restoration* was measured using the item, “How ‘back to normal’ do you think (child) would feel about his/her relationship with (transgressor) after what happened?” (“*not normal at all*” (unhappy face), “*a bit back to normal”* (slightly smiley face), “*totally back to normal*” (smiley face).

#### Socially Desirable Responding

*Socially desirable responding* was assessed using Short Form A of the Crandall Social Desirability Test for Children (CSDTC; Carifio, 1994), a 12-item forced-choice true-false measure of the tendency to give socially desirable or acceptable responses (α = 0.79).

### Procedure

Following University and Department of Education ethics approval, participants were recruited by the first author approaching schools, who forwarded study information and consent forms to parents/caregivers. Following informed parental consent, the study was run at schools with groups of between two and 10 participants. All materials were administered in English. No specific expertise is required to administer the CFCS tasks, other than ensuring the administrator has appropriate training in administering psychological tests, and is familiar with the protocol for this particular assessment tool.

At the beginning of each session, the researcher explained the voluntary nature of the study (including children's right to withdraw) and the anonymous and confidential nature of the data, and described the purpose of the study as finding out about how children feel, think and act when things go wrong between friends. Forgiveness was not mentioned by the researcher unless participants required the single-item forgiveness measure to be read aloud.

The researcher first read out the hypothetical scenario, with apology manipulated at group level such that one group received the apology manipulation and the next group received the no apology manipulation. Children then completed measures individually without communicating about their responses.

Children completed measures as follows: (1) background variables and manipulation checks; (2) forgiveness measures (CFCS, EFI-C and single-item explicit measure); (3) measure of relationship restoration; and (4) measure of socially desirable responding. The researcher read instructions for the CFCS and EFI-C aloud, and was available to help participants understand items or instructions at any time. Finally, although we did not time how long it took participants to complete the CFCS and the EFI-C, the impression of the first author—who administered the materials—is that participants took ~5 min to complete both the Sort and Line tasks of the CFCS and somewhat longer to complete the EFI-C.

## Results

### Part 1: The Psychometric Properties of the CFCS

#### Exploratory Factor Analysis of Card Set Scores

Initial analyses were conducted in R (R Core Team, 2015) using the “psych” package (Revelle, 2015) and the final solutions were estimated in MPlus v8.1 (Muthén and Muthén, 2018). The dimensionality of the two tasks was assessed by consideration of the eigen values, the scree test (Cattell, 1966), and a parallel roots analysis (Horn, 1965). Goodness of fit of EFA solutions was assessed using the chi-square statistic, the normed chi-square, the CFI, and the RMSEA (see, for example, Kline, 2011). For both the Sort Task and the Lines Task, there were two eigen values greater than unity and both the scree plot and parallel roots analysis suggested two factors be retained.

For the Sort Task, we specified the data as categorical (because scores could only take values of −1 or 1) and used a mean and variance adjusted weighted least squares estimator with pro-max rotation. The two-factor solution was not viable because there was only one item (the “weird/confused” card) with its highest loading on the second factor whereas the remaining items had very high loadings on the first factor. This item was therefore removed and the analysis was repeated. There was now only one eigen value greater than unity and both the scree plot and parallel roots analysis suggested a one factor solution. The one-factor solution fitted well [χ^2^_(90)_ = 106.8, *p* = 0.11 (normed chi-square = 1.19); CFI = 1.00; RMSEA = 0.04] and item loadings ranged from 0.87 to 0.98.

For the Line Task, we used maximum likelihood estimation with promax rotation. Examination of this solution again showed problems with the item “weird/confused.” The communality for this item was 0.06, whereas for the remaining items, the communalities ranged from 0.49 to 0.85 (*Md* = 0.74). As for the Sort Task, this item was therefore removed and the analysis was repeated; again a two factor solution was suggested and the fit for this solution was acceptable (χ^2^_(76)_ = 164.7, *p* < *0*.001, [normed chi-square = 2.17]; CFI = 0.96; RMSEA = 0.09). This solution is shown in Table 2. It may be seen that all eight positive cards and the “Upset” card loaded significantly on the first factor (*p* < 0.001), which was therefore interpreted as representing positivity (higher scores represented a more positive response, therefore positive loadings for the reverse-scored “Sad” card represent being less upset).

**Table 2:** Factor loadings and communalities for two-factor solution of fifteen items for the Line Task (Study 1).

**Card number**	**Card description**	**Factor 1**	**Factor 2**	***h*^**2**^**
16	Happy	**0.93**	−0.04	0.81
4	Joyful	**0.93**	−0.01	0.83
7	Warm	**0.79**	0.10	0.76
12	All OK/thumbs up	**0.77**	0.20	0.85
1	Upset	**0.71**	0.15	0.69
2	Playing/hanging out	**0.57**	0.31	0.69
6	Saying hi	**0.57**	0.33	0.70
15	Invite to play	**0.54**	0.32	0.64
10	Helping	**0.49**	0.34	0.59
13	“Get away from me”	−0.02	**0.88**	0.75
9	Hate	0.06	**0.87**	0.83
8	Not talking/listening	0.00	**0.82**	0.67
3	Fighting/arguing	0.15	**0.76**	0.77
5	Anger	**0.35**	**0.57**	0.74
11	Ignoring	0.19	**0.54**	0.49

*h^2^ are communalities; the correlation between the two factors is r = 0.73; loadings in bold p < 0.001*.

“Anger,” “Hatred,” and the four cards portraying negative behaviors loaded highest on the second factor, which was therefore interpreted as representing hostility (as negative cards were reverse scored, higher scores on this factor represented a more forgiving response, i.e., absence of hostility). While only the “Anger” card loaded significantly (*p* < 0.001) on both components, however it loaded more strongly on hostility (0.57) than positivity (0.35) and additionally it made most theoretical sense to retain in the hostility factor. It is noteworthy that four other items also had substantial cross-loadings (loadings > 0.3) without them reaching *p* < 0.001.

#### Reliability of the CFCS

Internal consistency was high for the Sort Task (α = 0.96, 15 items) and Line Task (α = 0.97, 15 items), and for positivity (α = 0.96, 9 items) and hostility (α = 0.93, 6 items) factors. Positivity and hostility scores correlated strongly with each other (*r* = 0.83, *p* < 0.01), and with the Sort Task (*r* = 0.88, *p* < 0.01 positivity; *r* = 0.88, *p* < 0.01 hostility) and Line Task (*r* = 0.97, *p* < 0.01 positivity; *r* = 0.94, *p* < 0.01 hostility); therefore, the Line Task was treated as a single score in subsequent analyses rather than analyzed according to these factors.

#### Relations Between the CFCS, Other Forgiveness Measures, and Socially Desirable Responding, and Age

We examined correlations between the CFCS Sort and Line Tasks and each of the EFI-C subscales, the single-item explicit forgiveness measure, and relationship restoration, as well as transgression severity, social desirability, and age (Table 3). As shown in Table 3, the CFCS Sort and Line Tasks correlated positively with all EFI-C subscales, the single-item explicit forgiveness measure, and relationship restoration, all at moderate levels with the exception of a slightly weaker correlation (= 0.26, <0.01) between the Sort Task and single-item explicit forgiveness. The Sort and Line Tasks correlated negatively with transgression severity. Neither CFCS task correlated significantly with socially desirable responding. Finally, the Line Task (but not the Sort Task) was negatively associated with age.

**Table 3:** Pearson Product Moment Correlation between CFCS Tasks, other forgiveness measures, severity, social desirability, and age (Study 1).

**Variable**	**1**	**2**	**3**	**4**	**5**	**6**	**7**	**8**	**9**
1. Line task									
2. Sort task	0.92^**^								
3. EFI-C feelings	0.42^**^	0.37^**^							
4. EFI-C behaviors	0.41^**^	0.36^**^	0.43^**^						
5. EFI-C thoughts	0.37^**^	0.35^**^	0.28^**^	0.67^**^					
6. Single-item forgiveness	0.34^**^	0.26^**^	0.32^**^	0.43^**^	0.49^**^				
7. Relationship restoration	0.33^**^	0.32^**^	0.20^*^	0.33^**^	0.40^**^	0.29^**^			
8. Severity	−0.31^**^	−0.25^**^	−0.35^**^	−0.14	−0.14	−0.15	−0.14		
9. Social desirability	0.16	0.10	0.09	0.00	0.05	−0.04	0.10	0.05	
10. Age	−0.20^*^	−0.16	−0.12	−0.01	0.01	0.15	0.08	0.07	−0.30^**^

*N = 112 for correlations with social desirability. N = 137 for correlations with age. All other Ns range from 145 to 148*.

* *p < 0.05*,

** *p < 0.01*.

#### Relations Between Gender and CFCS Tasks

A *t*-test indicated no significant differences between boys and girls on either the Sort Task [*t*_(143)_ = 0.01, *p* = 0.99, *d* = 0.00] or Line Task [*t*_(135)_ = 0.94, *p* = 0.35, *d* = 0.16].

#### Effect of Apology Manipulation

A *t*-test suggested the transgressor was judged to be significantly “more sorry” (greater perceived transgressor remorse) in the apology condition (*M* = 2.73, *SD* = 0.47) than in the no apology condition (*M* = 1.49, *SD* = 0.66) [*t*_(145)_ = 13.22, *p* < 0.01, *d* = s2.19]. Thus, the manipulation was successful.

Two separate *t*-tests were conducted with apology condition as the independent variable and the CFCS Sort and Line Tasks as dependent variables. For the Sort Task, there was no significant difference between the apology (*M* = −2.08, *SD* = 11.13), and no apology (*M* = −0.07, *SD* = 12.83) conditions, *t*_(143)_ = 1.01, *p* = 0.32, *d* = 0.17. Similarly for the Line Task, there was no significant difference between the apology (*M* = 69.41, *SD* = 43.10), and no apology (*M* = 76.96, *SD* = 50.52) conditions, *t*_(135)_ = 0.94, *p* = 0.35, *d* = 0.01. Thus, apology did not predict the Sort Task or the Line Task.

#### Incremental Effects of the Card Set on Relationship Restoration

We examined the extent to which CFCS tasks predicted unique variance on relationship restoration, over and above variance accounted for by the EFI-C subscales. We used relationship restoration as the dependent variable because it is considered a good behavioral indicator of forgiveness (e.g., McCullough, 2008).

We conducted two separate hierarchical regressions with apology condition entered as a control variable at step 1; the three EFI-C subscales entered at step 2; and each of the Sort and Line tasks entered separately at step 3. The Sort Task was a significant predictor of relationship restoration (β = *0*.20, *p* < 0.05, explaining an additional 3.0% of variance), as was the Line Task (β = 0.21, *p* < 0.05, explaining an additional 3.1% of variance).

### Part 2: Additional Analyses

The absence of an effect for apology on the CFCS Set Sort and Line Tasks led us to examine if this null finding was isolated to the CFCS or was also evident for the other forgiveness measures. Apology did not predict any of the EFI-C subscales or relationship restoration (all *p*s > 0.05), but did predict the single-item explicit forgiveness measure, *t*_(145)_ = 2.77, *p* = 0.01, *d* = 0.46.

In addition, examination of correlations between forgiveness measures and other variables (Table 3) identified age and severity as potential covariates due to correlation with CFCS scores. The assumption of independence of covariates (Field, 2009) was tested by conducting two separate analyses of variance with apology as the independent variable and age and severity as dependent variables; however, only severity met the assumption of independence from the apology condition *F*_(1, 145)_ = 0.43, *p* = 0.514, partial η^2^ = *0*.00. ANOVA was therefore repeated on CFCS scores with severity as a covariate; however, results were unaltered with no significant main effects found (*p*s > 0.05).

**Figure 2:**
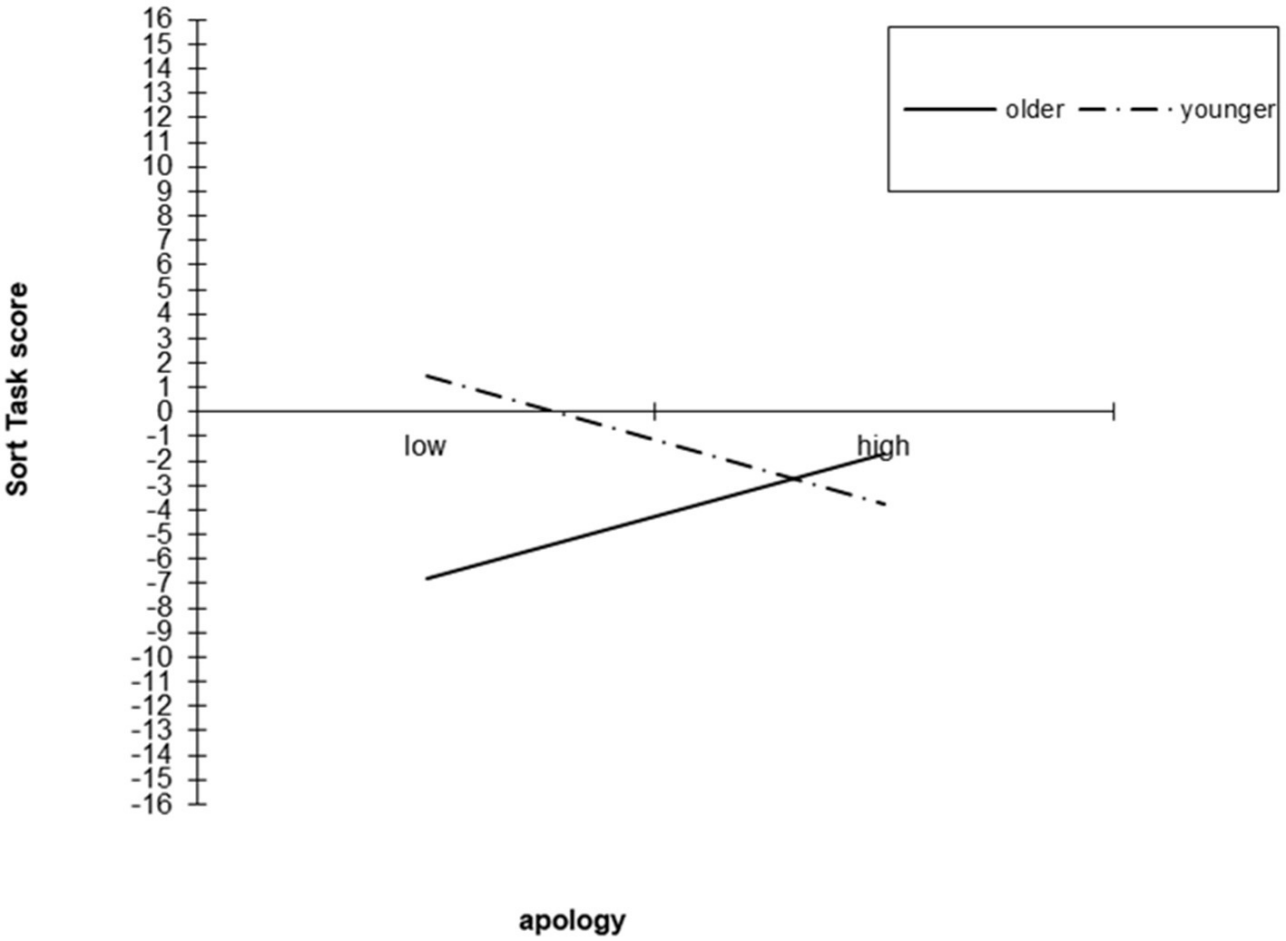
Interaction between apology and age in predicting the Sort Task, Study 1.

**Figure 3:**
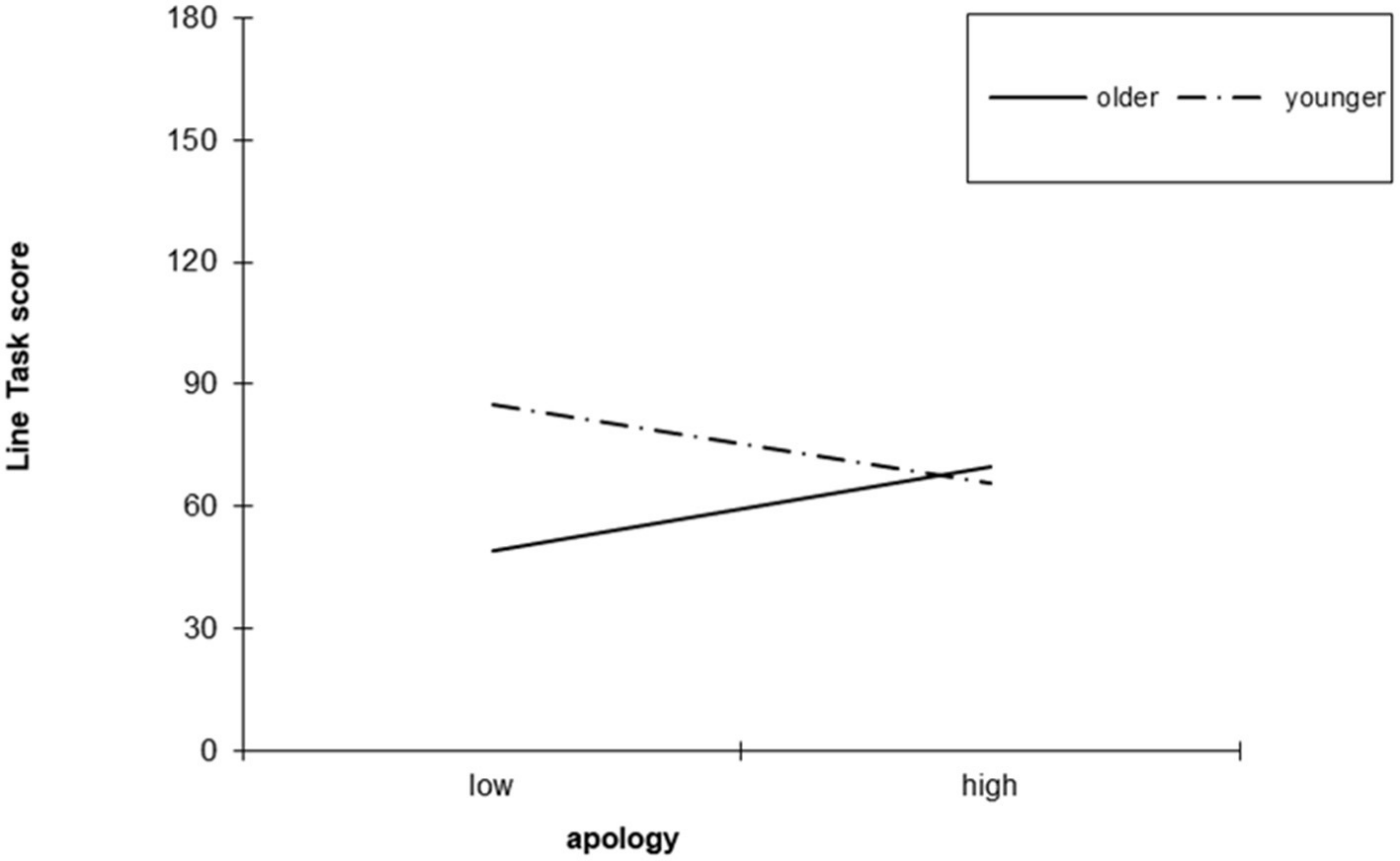
Interaction between apology and age in predicting the Line Task, Study 1.

Age did not meet the assumption of independence from the apology condition because participants were significantly older in the apology condition (*M* = 11.12, *SD* = 1.08) than in the no apology condition (*M* = 9.86, *SD* = 1.31); *F*_(1, 135)_ = 38.33, *p* > 0.01, partial η^2^ = 0.22. Therefore, the interaction between apology and age was examined in two ways. First, we conducted two separate hierarchical regression analyses with apology condition and mean-centered age entered at step 1 and the interaction between apology and mean-centered age entered at step 2. The interaction between age and apology was significant for the Sort Task (β = 0.27, *p* < 0.05, accounting for an additional 3.7% of variance) and for the Line Task (β = 0.27, *p* < 0.05, accounting for an additional 3.6% of variance). Inspection of interactions indicated older children tended to be less forgiving than younger children, and particularly unforgiving of transgressors who did not apologize, for both the Sort Task (Figure 2) and Line Task (Figure 3).

To examine this interaction more specifically, we ran an ANOVA with apology condition and age group [preadolescent: aged 8 to 11 years (*n* = 101) vs. adolescent: aged 12 to 13 years (*n* = 36)] as independent variables. The interaction between apology and age group had a significant effect on both the Sort Task *F*_(1, 130)_ = 6.43, *p* = 0.01, partial η^2^ = 0.047, and the Line Task *F*_(1, 130)_ = 8.43, *p* < 0.01, partial η^2^ = 0.07. Simple effects analysis indicated that for the Sort Task, there was no significant difference between adolescents and preadolescents in the apology condition, but in the no apology condition, adolescents (*M* = −9.86, *SD* = 11.01) were less forgiving than preadolescents (*M* = 0.71, *SD* = 12.65); *F*_(1, 130)_ = 5.09, *p* = 0.026, partial η^2^ = 0.038. Further, adolescents' scores were marginally higher in the apology condition (*M* = −0.29, *SD* = 10.01) than in the no apology condition (*M* = −9.86, *SD* = 11.01); *F*_(1, 130)_ = 3.76, *p* = 0.055, partial η^2^ = 0.028 whereas preadolescents' responses did not significantly differ across apology conditions (*p* > 0.05). For the Line Task, adolescents (*M* = 31.12, *SD* = 45.47) were again less forgiving than preadolescents (*M* = 82.09, *SD* = 48.35) in the no apology condition; *F*_(1, 122)_ = 7.89, *p* = 0.01, partial η^2^ = 0.061, but not in the apology condition. Additionally, adolescents were significantly more forgiving in the apology condition (*M* = 75.07, *SD* = 39.10) than in the no apology condition (*M* = 31.13, *SD* = 45.47); *F*_(1, 122)_ = 5.32, *p* = 0.023, partial η^2^ = 0.042, whereas preadolescents' responses did not significantly differ between apology and no apology conditions (*p* > 0.05).

### Study 2

Study 2 had five aims: (1) further test the factor structure of the CFCS; (2) test, again, the effect of manipulated apology on the CFCS; (3) expand the theoretical net to test whether manipulated ingroup salience also predicts the CFCS; (4) test whether the CFCS is amenable to online administration; and (5) test whether the factor structure and the apology effects observed in Study 1 among Australian children generalize to a sample of North American children.

A key function of forgiveness is that it helps restore and nourish valued relationships that are threatened by transgressions (Strelan et al., 2013). Relatedly, we earlier noted that an important feature of childhood developmental trajectories is the ability to build and maintain functional, healthy peer relations. Children are aware of ingroup/outgroup differences, even from an early age and even when groups are minimal (Dunham et al., 2011). Notably, children are more likely to judge transgressions against outgroup members as more acceptable than against ingroup members (Mulvey, 2016), and more likely to feel hurt by outgroup transgressors and more likely to forgive ingroup transgressors (Peets et al., 2013). Given robust historical and psychological evidence indicating that group membership affects levels of forgiveness and revenge among adults (e.g., Wenzel, 2019), Study 2 tested this factor in addition to apology.

#### Method

##### Participants

Participants were 154 children (aged 5 years to 12 years, *M* = 8.96 years, *SD* = 1.68, 91 Female). Children identified as 53.2% European-American, 20.8% African-American, 9.1% Latin, 5.9% Asian-American, 5.8% multiracial, and 5.3% other. Information about socio-economic status was not collected. Children were approached at a children's museum in the Southeastern United States and informed parental consent and child assent was obtained before participation. Each child received a small prize for participation. Note that the CFCS and the apology × ingroup salience manipulations were opportunistically employed in a testing session where children completed a battery of measures for an unrelated study comprising a larger sample of participants.

##### Procedures and Materials

Children sat with a research assistant and completed the study in a quiet space in the museum on a tablet computer. They could respond verbally or by touching the screen. For logistical reasons, it was only possible to present the CFCS Line Task. Other than reverse-coding negatively valenced cards, administration was as described in Study 1. The Line Task was measured on a scale where 0 = *would not feel like* to 10 = *would feel like*.

After completing unrelated familiarization and practice tasks, participants heard about two hypothetical peer groups (illustrated with same-gender outlines of five characters wearing all yellow or all green shirts and pants). They were introduced to their group, the Yellow Group, and completed a brief group affiliation task where they selected a name, an activity, and a symbol for their group (Mulvey and Killen, 2015). Next they were introduced to the other group, the Green Group. They were read a story in which a member of the Green (or Yellow) Groups tell an embarrassing story about them at school. The next day, the same child from the Green Group (or Yellow Group) said nothing about what they did yesterday and acted as nothing had happened (or the next day, they apologized, looked sad, and said they felt bad and would make up for what they did). Participants then completed the CFCS Line Task.

The manipulation check for apology was, “How sorry do you think the kid from the (Yellow or Green) Group felt about what happened?” (1 = *really sorry*; 6 = *not really sorry*). Finally, to test for age effects we categorized participants as younger (ages 7–9; 58% of the dataset) or older (ages 10–14; 42% of the data set).

There were missing data for each of the cards with sample sizes ranging from 146 to 154.

As for Study 1, we initially examined an EFA solution using maximum likelihood estimation with pro-max rotation. This solution is not reported here but again it was a two-factor solution and fitted moderately well hence demonstrating generalizability across different methods of administration and sample nationality. However, as for the solution reported in Table 2, there were a substantial number of cross-loadings. Taken together, the solutions for Studies 1 and 2 suggest the possibility that a better model for the Lines Task might be a bi-factor model whereby all cards load on a general forgiveness factor, and also on either a positive or negative factor; these two latter factors may prove to be correlated with each other but are independent of the general factor. Consequently, an exploratory bi-factor analysis (EBFA) with oblique rotation was employed. Developed by Jennrich and Bentler (2012), this method allows the identification of a bi-factor structure where all items load on the first, or general factor, and good cluster loadings for the remaining group factors, which are correlated with each other but not the general factor, are obtained.

#### Results

##### Exploratory Bi-Factor Analysis

An EBFA model was fitted in MPlus v8.1 (Muthén and Muthén, 2018) and Table 4 shows the results. The fit of this model was good: $\chi_{\left(63\right)}^{2}$ = 132.2, *p* < 0.001, CFI = 0.92, RMSEA = 0.08 with CI_90_ [0.06, 0.10], and SRMR = 0.05. This solution has several noteworthy features: (i) where loadings on the first general forgiveness factor are substantial (and statistically significant) they are in the expected direction, that is, positive for positivity and negative for hostility; (ii) for the group factors hostility and positivity, again the items behaved as would be expected; (iii) as is often the case with bi-factor solutions, some items had a high loading on the general factor and a lower loading on the relevant group factor (e.g., Card 1 “Upset” and Card 4 “Joyful”), while others better represented the relevant group factor rather than the general factor (e.g., Card 6 “Saying Hi,” Card 13 “Get Away from Me,” and Card 15 “Invite to Play”), and others measured both the general and relevant group factor about equally well (e.g., Card 2 “Playing/Hanging Out” and Card 3 “Fighting/Arguing”).

**Table 4:** Factor loadings exploratory bi-factor solution of fifteen items for the Line Task (maximum pairwise *N* = 154) (Study 2).

**Card number**	**Card description**	**Factor 1**	**Factor 2**	**Factor 3**
1	Upset	–**0.62**	0.20	0.07
2	Playing/hanging out	**0.42**	−0.07	**0.38**
3	Fighting/arguing	–**0.30**	**0.50**	0.06
4	Joyful	**0.75**	0.01	0.18
5	Anger	–**0.48**	**0.61**	0.01
6	Saying hi	0.13	−0.04	**0.71**
7	Warm	**0.59**	0.00	**0.31**
8	Not talking/listening	−0.16	**0.40**	−0.01
9	Hate	−0.14	**0.62**	–**0.22**
10	Helping	**0.37**	−0.10	**0.61**
11	Ignoring	–**0.38**	**0.69**	0.04
12	All OK/thumbs up	**0.49**	0.09	**0.53**
13	“Get away from me”	0.05	**0.75**	0.00
15	Invite to play	0.02	0.02	**0.76**
16	Happy	**0.65**	0.08	**0.42**

*Factor 1 is a general forgiveness factor, Factor 2 represents hostility and Factor 3 represents positivity. Factor 1 is orthogonal to both Factors 2 and 3 which in turn are correlated with each other at r = −0.27 (p < 0.05). Loadings in **bold** are p < 0.05*.

##### Effects of Apology and Group Membership on the CFCS

First, a two-way ANOVA indicated that the apology manipulation was successful. Participants in the apology condition (*M* = 4.30; *SD* = 1.42) were more likely than those in the no apology condition (*M* = 3.30, *SD* = 1.69) to indicate that the transgressing child was sorry, *F*_(1, 150)_ = 16.83, *p* < 0.001, partial η^2^ = 0.101. There was no difference between the in and out groups (*p* = 0.060) nor an interaction (*p* = 0.860).

Next we tested whether apology and group membership predicted the CFCS. A two-way ANOVA revealed that participants in the apology condition (*M* = 4.29, *SD* = 2.63) scored higher than those in the no apology condition (*M* = 3.40, *SD* = 2.10) on the Positive Card Set, *F*_(1, 135)_ = 5.50, *p* = 0.021, partial η^2^ = 0.039. There was no difference between the ingroup (*M* = 4.04, *SD* = 2.39) and outgroup (*M* = 3.60, *SD* = 2.26) conditions on the Positive Card Set, *F*_(1, 135)_ = 1.68, *p* = 0.197. The interaction was non-significant, *F*_(1, 135)_ = 0.56, *p* = 0.814.

There was also a significant difference between the apology conditions (*M* = 4.59, *SD* = 2.20 vs. *M* = 5.36, *SD* = 2.30), *F*_(1, 134)_ = 4.27, *p* = 0.041, partial η^2^ = 0.031 on the Negative Card Set, with participants in the apology condition scoring lower. There was no effect of group membership on the Negative Card Set (*in*group *M* = 5.00, *SD* = 2.26 vs. *out*group *M* = 4.98, *SD* = 2.27), *F*_(1, 134)_ = 0.00, *p* = 0.995, nor an interaction, *F*_(1, 134)_ = 1.63, *p* = 0.204.

##### Supplementary Analyses

Because we found a significant apology x age interaction on the Card Set in Study 1, we conducted an additional analysis, adding age into a 2 (apology) × 2 (group) × 2 (age) ANOVA. The apology × age interaction on the Positive Card Set was non-significant, *F*_(1, 131)_ = 1.02, *p* = 0.313, and also non-significant for the Negative Card Set, *F*_(1, 130)_ = 2.16, *p* = 0.144.

## Discussion

### Part 1: The Psychometric Properties of the CFCS

Exploratory factor analysis (Study 1) established 15 cards in the final CFCS, along with positivity and hostility components for the Line Task but a single factor for the Sort Task. Exploratory factory analysis (Study 2, solution not reported here) confirmed the two-factor structure (positivity and hostility) of the Line Task. Notably, the Line Task structure generalizes across Australian (Study 1) and US (Study 2) children and also across traditional hard copy presentation (Study 1) and electronic administration (Study 2). Across both samples, internal consistency reliability was high. In Study 1, which also tested concurrent validity, the CFCS correlated consistently with all other forgiveness measures, at mostly moderate levels. As such, the CFCS appears a potentially valid measure of children's underlying responses to transgression.

Scrutiny of EFA solutions in both Study 1 and Study 2 led to the fitting of an EFBA model to the data for Study 2. This solution has interesting features and allows the possibility of using a reduced set of cards to measure either general forgiveness, or to focus on positivity, or hostility, or both of them.

In terms of incremental validity, to what extent is the CFCS a useful addition to existing measures? In particular, the EFI-C might arguably be just as useful for assessing children's forgiving emotions. However, the CFCS correlated with EFI-C subscales at only moderate levels, and regression analysis suggested the CFCS predicted a small but significant amount of unique variance in predicting relationship restoration. Additionally, because the CFCS is based on feelings and behaviors identified by children as being involved in forgiveness, it is unique as a child-focused measure, rather than adapted downward from an adult measure. Finally, the observations of the researcher administering Study 1 was that children appeared to enjoy sorting the cards, while adolescents appeared to prefer the questionnaire. The CFCS may therefore be considered particularly appropriate to preadolescent children and may be especially useful for children who do not engage well in reading or listening tasks.

One objective of the CFCS was to avoid the use of response scales by employing a sorting task. However, a line was added to assess the strength of children's responses, producing alternative Sort Task and Line Task scores. These were highly correlated and produced similar results; slight exceptions were that the Sort Task produced only one component and correlated with other forgiveness measures at slightly lower levels than the Line Task. Overall, the Sort and Line Tasks appear comparable. The Sort Task may therefore be sufficient, particularly for younger children who may find the Line Task difficult; however, the Line Task may remain relevant when a more sensitive score is preferred.

Ultimately, whether the CFCS is a valid measure of children's forgiveness depends upon how forgiveness is defined. If a researcher is interested primarily in emotional reactions to transgression, the CFCS appears to measure these reactions reasonably sensitively.

### Part 2: Theoretical and Practical Implications of the CFCS for Children's Forgiveness

The CFCS is conceptualized as a measure of transgression-specific forgiveness, although it would also be easily adapted as a trait measure (e.g., by asking participants to think about how they generally respond to transgressions and respond to the Sort and Line Tasks accordingly). Interestingly, our two samples yielded mixed results when it came to transgression-specific predictors of the CFCS. First, manipulating group membership did not predict the Line Task in Study 2. Second, on one hand, mirroring extant child and adult research, apology predicted the CFCS Line Task in Study 2. On the other hand, in Study 1 the effect of apology on the Sort and Line Tasks was contingent upon age, with older children more likely to forgive. Further, the absence of a main effect for apology in Study 1 was not confined to the CFCS; apology also did not predict the EFI-C subscales. Given the geographical and administration differences between the two samples (Australian vs. US; hard copy vs. online), it is difficult to pinpoint likely explanations for the differences in findings for apology in particular—and also moderating effects of age—suffice to say that more research is required to identify the extent to which transgression-specific variables predict the CFCS.

#### Emotional and Decisional Forgiveness

The apparent difference between children's latent responses and explicit responses is congruent with theoretical distinctions between emotional forgiveness, which is multifaceted and involves changes in emotion, cognition and motivation and eventually behavior, and decisional forgiveness, which is a decision to control one's behaviors (e.g., Worthington et al., 2007). Such distinctions are important because the two processes are likely to have different consequences for well-being. For example, Worthington and colleagues postulate that while decisional forgiveness can be a permanent and sincere form of forgiving which may reduce outward hostility, it does not necessarily reduce internalized stress responses. Likewise, Karremans and Van Lange (2008) argue that although forgiveness is often viewed as deliberative and intentional, the decision to forgive does not necessarily result in the dissipation of negative feelings. In contrast, emotional forgiveness has a stronger connection to overcoming negative affect and stress responses, and is more likely to have a direct influence on individual health (Worthington et al., 2007).

To the extent that children's explicit judgments of forgiveness can be seen to represent their decisional forgiveness, differential prediction of their explicit and latent responses in Study 1 suggests children may experience both underlying/emotional forgiveness (i.e., responses on the CFCS and EFI-C subscales) and decisional forgiveness (i.e., responses on the single-item explicit measure). Moreover, the impact of situational variables (such as apology) may sometimes differ between these different types of forgiveness. Therefore, future research examining predictors of children's forgiveness may need to specify the type of forgiveness assessed (i.e., emotional/decisional, underlying/explicit), and may compare the impact of situational variables across different types of forgiveness.

Differences between children's explicit and emotional forgiveness may also have potential practical implications. Negative emotional responses to a transgressor may continue regardless of apology, and children who say they have forgiven may potentially continue to experience emotional hurt related to the transgression. Considering that children are aware of the “moral goodness” of forgiveness but also value sincerity in forgiving (Kemp et al., 2009), adults should refrain from unrealistic expectations of children's forgiveness necessarily healing emotional hurt caused by transgression may cause. This is particularly the case when forgiveness is suggested for such uses as coping with school bullying (e.g., Egan and Todorov, 2009), as it is important that children do not feel persecuted or disempowered by their decision either to forgive or not.

Overall, distinctions between underlying and explicit forgiveness are such that a measure of children's underlying emotional responses such as the CFCS is a potentially important tool in examining precursors and consequences of children's forgiveness.

### Limitations and Directions for Future Research

The CFCS provides a measure of *transgression-specific* forgiveness. The adult literature on forgiveness clearly identifies several key transgression-specific variables that predict forgiveness, including perceived intent, severity, and relationship closeness [for a meta-analysis, see Fehr et al. (2010)]. We tested two, apology (in both studies) and group membership (Study 2). Thus, first, to further confirm the validity of the CFCS, there is now a need to examine the extent to which it is predicted by other salient indicators of transgression-specific forgiveness. Relatedly, future researchers could test the extent to which trait-level variables and relevant individual differences predict responses on the CFCS. Attachment, for example, with its roots in infant and early childhood experiences, has been shown to predict adult willingness to forgive (e.g., Burnette et al., 2009). Similarly, on the basis that some early childhood experiences can be traumatic and can affect ability to forgive (e.g., Mucci, 2018), future studies may consider the potential direct or moderating effect of trauma.

Second, although hypothetical methodology is in keeping with previous research on children's forgiveness, responses to a hypothetical scenario may differ to responses to a real life transgression. This limitation may particularly apply to differences between younger and older children's CFCS responses; given hypothetical transgressions require more perspective-taking ability than personally experienced transgressions (Smith and Harris, 2012), younger children may not have been able to realistically imagine the victim's emotional responses, even if they could give the “expected” response to apology in terms of explicit and cognitive responses. Further study would benefit from applying the CFCS to personally experienced transgressions.

Third, the impact of the passage of time on forgiveness was not explored. Children responded directly after hearing the scenario and forgiveness was assessed at only one time point; however because forgiveness refers to change over time, such a cross-sectional approach, while common in forgiveness research, may not fully assess changes across the forgiveness trajectory (McCullough et al., 2003). Relatedly, forgiveness is a process (for a review, see Strelan, 2019). Several therapeutic interventions have been developed which enable adult clients to work through the forgiveness process (for a review see Wade et al., 2014). However, interventions suitable for children are yet to be developed. The CFCS could potentially be utilized to capture children's level of forgiveness in therapeutic interventions.

A fourth limitation is that validity in terms of correlation with other constructs that can be expected to relate to forgiveness, such as co-operative behavior, are yet to be examined; likewise discriminant validity was only examined with respect to a measure of socially desirable responding and ideally will be examined in relation to other constructs in further study.

Fifth, our studies were conducted with children from two Westernized societies. Although the universality of the forgiveness construct among adults is established (e.g., Karremans et al., 2011), nonetheless there is a need to test the extent to which the CFCS resonates with children from non-Westernized countries.

Finally, the present data indicate that the Line and Sort scores are highly correlated (Study 1) and associated with other forgiveness-relevant measures to a similar degree. However, our experience in Study 1 during the administration phase was that children tended to complete the Sort task quicker, and younger children found the sort requirements easier to follow, consistent with our aim that the CFCS provide a measure of children's forgiveness without having to rely on children's linguistic competence. Nonetheless, more research is required so that a definitive recommendation can be made about the utility of the Card Sort task relative to the Line task.

## Conclusion

These two initial studies suggest the CFCS may be an effective way for children to report emotional responses to transgression without verbal or written reporting. As a measure of emotion-based reactions to transgression, the CFCS has potential as a valid, reliable and useful measure that may have practical advantages in addressing children's underlying emotional reactions even in cases in which they explicitly report having “forgiven.” As such, the CFCS could be used for a range of applications, including clinical use (e.g., to assess emotional responses to a transgressor), school-based interventions concerning peer relations or bullying, or use with non-verbal children who find language and articulation difficult.

## Data Availability Statement

The datasets presented in this study can be found in online repositories. The names of the repository/repositories and accession number(s) can be found at: https://adelaide.figshare.com/articles/dataset/_/13193789.

## Ethics Statement

The studies involving human participants were reviewed and approved by University of Adelaide (Study 1) and North Carolina State University (Study 2). Written informed consent to participate in this study was provided by the participants' legal guardian/next of kin.

## Author Contributions

EK developed the card set, collected the data for Study 1, analyzed the data for Study 1, and was responsible for the majority writing of the paper. PS supervised the project, assisted in analyzing data and writing up. RR supervised the project and read drafts. NB conducted and wrote up the factor analyses. KM developed and conducted Study 2. All authors contributed to the article and approved the submitted version.

## Conflict of Interest

The authors declare that the research was conducted in the absence of any commercial or financial relationships that could be construed as a potential conflict of interest.

## References

[B1] Anthony E. J. Bene E. (1957). A technique for the objective assessment of the child's family relationships. Br. J. Psychiatry 103, 541–555. 10.1192/bjp.103.432.541 13449562

[B2] Bagwell C. L. Newcomb A. F. Bukowski W. M. (1998). Preadolescent friendship and peer rejection as predictors of adult adjustment. Child Dev. 69, 140–153. 10.1111/j.1467-8624.1998.tb06139.x 9499563

[B3] Barker J. Weller S. (2003). “Is it fun?” Developing child centred research methods. Int. J. Sociol. Soc. Policy 23, 33–58. 10.1108/01443330310790435

[B4] Bradfield M. Aquino K. (1999). The effects of blame attributions and offender likableness on forgiveness and revenge in the workplace. J. Manage. 25, 607–631. 10.1177/014920639902500501

[B5] Burnette J. L. Davis D. E. Green J. D. Worthington E. L. Bradfield E. (2009). Insecure attachment and depressive symptoms: the mediating role of rumination, empathy, and forgiveness, Pers. Individ. Differ. 46, 276–280, 10.1016/j.paid.2008.10.016

[B6] Carifio J. (1994). Sensitive data and students' tendencies to give socially desirable responses. J. Alcohol Drug Educ. 39, 74–84.

[B7] Carlisle R. D. Tsang J. Ahmad N. Y. Worthington E. L. Jr. Witvliet C. V. O. Wade N. (2012). Do actions speak louder than words? Differential effects of apology and restitution on behavioral and self-report measures of forgiveness. J. Positive Psychol. 7, 294–305. 10.1080/17439760.2012.690444

[B8] Cattell R. B. (1966). The scree test for the number of factors. Multivariate Behav. Res. 1, 245–276. 10.1207/s15327906mbr0102_10 26828106

[B9] Chambers C. T. Johnston C. (2002). Developmental differences in children's use of rating scales. J. Pediatr. Psychol. 27, 27–36. 10.1093/jpepsy/27.1.27 11726677

[B10] Christensen K. J. Padilla-Walker L. M. Busby D. M. Hardy S. A. Day R. D. (2011). Relational and social-cognitive correlates of early adolescents' forgiveness of parents. J. Adolesc. 34, 903–913. 10.1016/j.adolescence.2011.01.001 21296401

[B11] Darby B. W. Schlenker B. R. (1982). Children's reactions to apologies. J. Pers. Soc. Psychol. 43, 742–753. 10.1037/0022-3514.43.4.742

[B12] Denham S. A. Bouril B. (1994). Preschooler's affect and cognition about challenging peer situations. Child Study J. 24, 1–22.

[B13] Denham S. A. Neal K. Wilson B. J. Pickering S. Boyatzis C. J. (2005). Emotional development and forgiveness in children: emerging evidence, in Handbook of Forgiveness, eds Worthington E.L. Jr. (New York, NU: Brunner-Routledge), 127-142.

[B14] Dunham Y. Baron A. S. Carey S. (2011). Consequences of 'minimal' group affiliations in children. Child Dev. 82, 793–811. 10.1111/j.1467-8624.2011.01577.x 21413937 PMC3513287

[B15] Egan L. A. Todorov N. (2009). Forgiveness as a coping strategy to allow school students to deal with the effects of being bullied: theoretical and empirical discussion. J. Soc. Clin. Psychol. 28, 198–222. 10.1521/jscp.2009.28.2.198

[B16] Eiser C. Mohay H. Morse R. (2000). The measurement of quality of life in young children. Child Care Health Dev. 26, 401–414. 10.1046/j.1365-2214.2000.00154.x 10998003

[B17] Enright R. D. (2000). The Enright Forgiveness Inventory for Children. Madison WI: Department of Educational Psychology, University of Wisconsin-Madison.

[B18] Enright R. D. Santos M. J. Al-Mabuk R. (1989). The adolescent as forgiver. J. Adolesc. 12, 9–110. 10.1016/0140-1971(89)90092-4 2708604

[B19] Fattore T. Mason J. Watson E. (2007). Children's conceptualisation(s) of their well-being. Soc. Indic. Res. 80, 5–29. 10.1007/s11205-006-9019-9

[B20] Fehr R. Gelfand M. J. (2010). When apologies work: how matching apology components to victims' self-construals facilitates forgiveness. Organ. Behav. Hum. Decis. Process 13, 37–50. 10.1016/j.obhdp.2010.04.002

[B21] Fehr R. Gelfand M. J. Nag M. (2010). The road to forgiveness: a meta-analytic synthesis of its situational and dispositional correlates. Psychol. Bull. 136, 894–914. 10.1037/a0019993 20804242

[B22] Field A. (2009). Discovering Statistics Using SPSS (3rd edition). Sage: London.

[B23] Goldring J. Strelan P. (2017). The forgiveness implicit association test. Pers. Individ. Dif, 108, 69–78. 10.1016/j.paid.2016.12.006

[B24] Greenwald A. G. Banaji M. R. (1995). Implicit social cognition: attitudes, self-esteem, and stereotypes. Psychol. Rev. 102, 4–27. 10.1037/0033-295X.102.1.4 7878162

[B25] Horn J. L. (1965). A rationale and test for the number of factors in factor analysis. Psychometrika 30, 179–185. 10.1007/BF02289447 14306381

[B26] Hoyt W. T. McCullough M. E. (2005). Issues in the multi-modal measurement of forgiveness, in Handbook of Forgiveness, ed Worthington E.L. Jr. (New York, NY: Brunner-Routledge), 109–123.

[B27] Hui E. K. P. Chau T. S. (2009). The impact of a forgiveness intervention with Hong Kong Chinese children hurt in interpersonal relationships. Br. J. Guidance Counselling 37, 141–156. 10.1080/03069880902728572

[B28] Jennrich R. I. Bentler P. M. Exploratory Bi-factor analysis: The oblique case. Psychometrika (2012) 77, 442–454. 10.1007/s11336-012-9269-1 27519775

[B29] Karremans J. C. Aarts H. (2007). The role of automaticity in determining the inclination to forgive close others. J. Exp. Soc. Psychol. 43, 902–917. 10.1016/j.jesp.2006.10.012

[B30] Karremans J. C. Regalia C. Paleari F. G. Fincham F. D. Cui M. Takada N.. (2011). Maintaining harmony across the globe: the cross-cultural association between closeness and interpersonal forgiveness. Soc. Psychol. Personal. Sci. 2, 443–451. 10.1177/1948550610396957

[B31] Karremans J. C. Van Lange P. A. M. (2008). Forgiveness in personal relationships: Its malleability and powerful consequences. Eur. Rev. Soc. Psychol. 19, 202–241. 10.1080/10463280802402609

[B32] Kazdin A. E. Petti T. A. (1982). Self-report and interview measures of childhood and adolescent depression. J. Child Psychol. Psychiatry 23, 437–457. 10.1111/j.1469-7610.1982.tb00089.x 7130301

[B33] Kemp E. B. Strelan P. Roberts R. (2009). The development of children's forgiveness: Children's understandings of forgiveness. Paper presented at the 16^th^ Biennial Australian Human Development Association Conference.

[B34] Kline R. (2011). Principles and Practice of Structural Equation Modeling (3rd ed.). New York, NY: Guilford Press.

[B35] Lawler-Row K. A. Scott C. Raines R. L. Edlis-Matityahou M. Moore E. W. (2007). The varieties of forgiveness experience: working toward a comprehensive definition of forgiveness. J. Relig. Health 46, 233–248. 10.1007/s10943-006-9077-y

[B36] Luby J. L. Svrakic D. M. McCallum K. Przybeck T. R. Cloninger C. R. (1999). The junior temperament and character inventory: preliminary validation of a child self-report measure. Psychol. Rep. 84, 1127–1138. 10.2466/pr0.1999.84.3c.1127 10477935

[B37] McCullough M. E. (2008). Beyond Revenge: The Evolution of the Forgiveness Instinct. San Francisco, CA: Jossey-Bass.

[B38] McCullough M. E. Fincham F. D. Tsang J. (2003). Forgiveness, forbearance, and time: the temporal unfolding of transgression-related interpersonal motivations. J. Pers. Soc. Psychol. 84, 540–557. 10.1037/0022-3514.84.3.540 12635915

[B39] McCullough M. E. Pargament K. I. Thoresen C. E. (2000). The psychology of forgiveness: history, conceptual issues, and overview, in Forgiveness: Theory, Research and Practice, eds McCullough M.E. Pargament K.I. Thoresen C.E. (New York, NY: Guilford Press), 1–14.

[B40] McCullough M. E. Worthington E. L. Jr Rachal K. C. (1997). Interpersonal forgiving in close relationships. J. Pers. Soc. Psychol. 73, 321–336. 10.1037/0022-3514.73.2.321 9248052

[B41] Mucci C. (2018). Beyond Individual and Collective Trauma. New York, NY: Routledge.

[B42] Mulvey K. L. (2016). Children's reasoning about social exclusion: balancing many factors. Child Dev. Perspect. 10, 22–27. 10.1111/cdep.12157

[B43] Mulvey K. L. Killen M. (2015). Challenging gender stereotypes: resistance and exclusion. Child Dev. 86, 681–694. 10.1111/cdev.12317 25382748

[B44] Muthén B. Muthén L. (2018). Mplus Version 8: User's Guide. Los Angeles, CA: Muthén and Muthén.

[B45] Oostenbroek J. Vaish A. (2019). The emergence of forgiveness in young children. Child Dev. 90, 1969–1986. 10.1111/cdev.13069 29607484

[B46] Peets K. Hodges E. V. Salmivalli C. (2013). Forgiveness and its determinants depending on the interpersonal context of hurt. J. Exp. Child Psychol. 114, 131–145. 10.1016/j.jecp.2012.05.009 22784854

[B47] R Core Team (2015). R: A Language and Environment for Statistical Computing. R Foundation for Statistical Computing, Vienna, Austria. Available online at: https://www.R-project.org/.

[B48] Rennik J. E. McHarg L. F. Dell'Api N. Johnston C. C. Stevens B. (2008). Developing the children's critical illness impact scale: capturing data from children, parents, and staff. Pediatric Critical Care Med. 9, 252–260. 10.1097/PCC.0b013e31816c70d4 18446107

[B49] Revelle W. (2015). Psych: Procedures for Personality and Psychological Research. Available online at: http://CRAN.R-project.org/package=psych, Version = 1.5.6.

[B50] Rizkalla L. Wertheim E. H. Hodgson L. K. (2008). The roles of emotion management and perspective taking in individuals' conflict management styles and disposition to forgive. J. Res. Pers. 42, 1594–1601. 10.1016/j.jrp.2008.07.014

[B51] Smith C. E. Chen D. Harris P. L. (2010). When the happy victimizer says sorry: children's understanding of apology and emotion. Br. J. Dev. Psychol. 28, 727–746. 10.1348/026151009X475343 21121464

[B52] Smith C. E. Harris P. L. (2012). He didn't want me to feel sad: children's reactions to disappointment and apology. Soc. Dev. 21, 215–228. 10.1111/j.1467-9507.2011.00606.x

[B53] Soto C. J. John O. P. Gosling S. D. Potter J. (2008). The developmental psychometrics of Big Five self-reports: acquiescence, factor structure, coherence, and differentiation from ages 10 to 20. J. Pers. Soc. Psychol. 94, 718–737. 10.1037/0022-3514.94.4.718 18361680

[B54] Strelan P. (2018). Justice and forgiveness in interpersonal relationships. Curr. Dir. Psychol. Sci. 27, 20–24. 10.1177/0963721417734311

[B55] Strelan P. (2019). The stress-and-coping model of forgiveness: theory, research, and the potential of dyadic coping, in Handbook of Forgiveness (2nd ed.), eds Worthington E. L. Wade N. G. (New York, NY: Routledge), 63–73. 10.4324/9781351123341-7

[B56] Strelan P. Crabb S. Chan D. Jones L. (2017). Lay perspectives on the costs and risks of forgiving. Pers. Relatsh 24, 392–407. 10.1111/pere.12189

[B57] Strelan P. McKee I. Calic D. Cook L. Shaw L. (2013). For whom do we forgive? A functional analysis of forgiveness. Personal Relationships 20, 124–139. 10.1111/j.1475-6811.2012.01400.x

[B58] van der Wal R. C. Karremans J. C. Cillesen A. H. (2016). Interpersonal forgiveness and psychological well-being in late childhood. Merrill Palmer Q 62, 1–21. 10.13110/merrpalmquar1982.62.1.0001

[B59] Wade N. G. Hoyt W. T. Kidwell J. E. M. Worthington E. L. (2014). Efficacy of psychotherapeutic interventions to promote forgiveness. a meta-analysis. J. Consulting Clin. Psychol. 82, 154–170. 10.1037/a0035268 24364794

[B60] Wainryb C. Recchia H. Faulconbridge O. Pasupathi M. (2019). To err is human: forgiveness across childhood and adolescence. Soc. Dev. 29, 509–525. 10.1111/sode.12413

[B61] Wenzel M. (2019). Forgiveness, reconciliation, and peace between groups, in Handbook of Forgiveness (2nd ed.), eds Worthington E. L. Wade N. G. (New York, NY: Routledge), 322–332. 10.4324/9781351123341-30

[B62] Worthington E. L. Jr. (2001). Five Steps to Forgiveness: The Art and Science of Forgiving. New York, NY: Crown.

[B63] Worthington E. L. Jr. (2006). The development of forgiveness, in Encyclopedia of Religious and Spiritual Development, eds Dowling E.M. Scarlett W.G. (Thousand Oaks, CA: Sage), 165–167.

[B64] Worthington E. L. Jr. Wade N. G. (2019). A new perspective on forgiveness research, in Handbook of Forgiveness (2nd ed.), eds Worthington E. L. Wade N. G. (New York, NY: Routledge), 345–355. 10.4324/9781351123341-32

[B65] Worthington E. L. Jr. Witvliet C. V. O. Pietrini P. Miller A. J. (2007). Forgiveness, health, and well-being: a review of evidence for emotional versus decisional forgiveness, dispositional forgivingness, and reduced unforgiveness. J. Behav. Med. 30, 291–302. 10.1007/s10865-007-9105-8 17453329

